# Pulmonary diffusing capacity to nitric oxide and carbon monoxide during exercise and in the supine position: a test–retest reliability study

**DOI:** 10.1113/EP090883

**Published:** 2023-01-09

**Authors:** Anna Christrup Madsen, Rie Skovly Thomsen, Stine B. Nymand, Jacob Peter Hartmann, Iben E. Rasmussen, Milan Mohammad, Lene Theil Skovgaard, Birgitte Hanel, Simon Jønck, Ulrik Winning Iepsen, Regitse H. Chistensen, Jann Mortensen, Ronan M. G. Berg

**Affiliations:** ^1^ Centre for Physical Activity Research Copenhagen University Hospital – Rigshospitalet Copenhagen Denmark; ^2^ Department of Biomedical Sciences Faculty of Health and Medical Sciences University of Copenhagen Copenhagen Denmark; ^3^ Department of Clinical Physiology and Nuclear Medicine Copenhagen University Hospital – Rigshospitalet Copenhagen Denmark; ^4^ Department of Biostatistics Faculty of Health and Medical Sciences University of Copenhagen Copenhagen Denmark; ^5^ Department of Anaesthesiology and Intensive Care Copenhagen University Hospital – Bispebjerg Hospital Copenhagen Denmark; ^6^ Department of Clinical Medicine Faculty of Health and Medical Sciences University of Copenhagen Copenhagen Denmark; ^7^ Department of Cardiology Copenhagen University Hospital – Herlev and Gentofte Hospitals Copenhagen Denmark; ^8^ Neurovascular Research Laboratory Faculty of Life Sciences and Education University of South Wales Pontypridd UK

**Keywords:** alveolar–capillary reserve, pulmonary gas exchange, repeatability

## Abstract

*D*_LCO/NO_, the combined single‐breath measurement of the diffusing capacity to carbon monoxide (*D*_LCO_) and nitric oxide (*D*_LNO_) measured either during exercise or in the resting supine position may be a useful physiological measure of alveolar–capillary reserve. In the present study, we investigated the between‐day test–retest reliability of *D*_LCO/NO_‐based metrics. Twenty healthy volunteers (10 males, 10 females; mean age 25 (SD 2) years) were randomized to repeated *D*_LCO/NO_ measurements during upright rest followed by either exercise (*n* = 11) or resting in the supine position (*n* = 9). The measurements were repeated within 7 days. The smallest real difference (SRD), defined as the 95% confidence limit of the standard error of measurement (SEM), the coefficient of variance (CV), and intraclass correlation coefficients were used to assess test–retest reliability. SRD for *D*_LNO_ was higher during upright rest (5.4 (95% CI: 4.1, 7.5) mmol/(min kPa)) than during exercise (2.7 (95% CI: 2.0, 3.9) mmol/(min kPa)) and in the supine position (3.0 (95% CI: 2.1, 4.8) mmol/(min kPa)). SRD for *D*_LCOc_ was similar between conditions. CV values for *D*_LNO_ were slightly lower than for *D*_LCOc_ both during exercise (1.5 (95% CI: 1.2, 1.7) vs. 3.8 (95% CI: 3.2, 4.3)%) and in the supine position (2.2 (95% CI: 1.8, 2.5) vs. 4.8 (95% CI: 3.8, 5.4)%). *D*_LNO_ increased by 12.3 (95% CI: 11.1, 13.4) and *D*_LCOc_ by 3.3 (95% CI: 2.9, 3.7) mmol/(min kPa) from upright rest to exercise. The *D*_LCO/NO_ technique provides reliable indices of alveolar–capillary reserve, both during exercise and in the supine position.

## Abstract (highlights)

- **What is the central question in this study?**
  How reliable is the combined measurement of the pulmonary diffusing capacity to carbon monoxide and nitric oxide (*D*_LCO/NO_) during exercise and in the resting supine position, respectively?
- **What is the main finding and its importance?**
  The *D*_LCO/NO_ technique is reliable with a very low day‐to‐day variability both during exercise and in the resting supine position, and may thus provide a useful physiological outcome that reflects the alveolar–capillary reserve in humans.

## 1 INTRODUCTION

Since the inception of pulmonary diffusing capacity (*D*_L_) measurements in humans more than a century ago (Bohr, 1909; Krogh, 1915; Krogh & Krogh, 1910), the impact of changes in pulmonary gas exchange on exercise capacity has been debated (Borland & Hughes, 2020; Hsia, 2002). In the healthy upright lung, gas exchange occurs across only a small fraction of the alveolar–capillary interface during rest, because less than half of the pulmonary capillaries are perfused (Okada et al., 1994, 1992; Warrell et al., 1972). The recruitment and distension of pulmonary capillaries triggered by an increase in cardiac output and central blood volume give rise to the so‐called alveolar–capillary reserve, which allows pulmonary diffusing capacity to increase appropriately during exercise (Hsia, 2002).

Recent studies have highlighted *D*_L_ measured during exercise as a potentially clinically relevant outcome reflecting the extent of pulmonary vascular (mal)adaptation in patients with chronic obstructive pulmonary disease (COPD) (Ross et al., 2020; Tedjasaputra et al., 2018). Uniquely, these studies measured *D*_L_ to carbon monoxide (*D*_LCO_) using the multiple fractions of inspired oxygen ($mF_{IO_{2}}$) technique permitting the quantitation of the components that together determine the rate of diffusion across the alveolar–capillary interface, and thus the alveolar–capillary reserve. These consist of the membrane diffusing capacity (*D*_M_), depending on the area and thickness of the alveolar–capillary interface available for gas exchange, and the pulmonary capillary blood volume (*V*_c_) (Roughton & Forster, 1957).

A recurring challenge when using the $mF_{IO_{2}}$ technique during exercise is that several manoeuvres must be performed at different $F_{IO_{2}}$ levels with repeated breath‐holds, which may be particularly difficult at relatively high exercise intensities, when studying patients with exertional dyspnoea and related exercise‐induced symptoms as in COPD. An alternative method for measuring *D*_L_ that may be used to assess the alveolar–capillary reserve is the combined single‐breath measurement of *D*_LCO_ and nitric oxide (*D*_LNO_), the so‐called *D*_LCO/NO_ technique (Munkholm et al., 2018; Zavorsky et al., 2017). Like the $mF_{IO_{2}}$ technique, *D*_LCO/NO_ permits the quantification of *D*_M_ and *V*_c_, but it is less technically demanding, mainly because it requires fewer manoeuvres (Borland & Hughes, 2020; Munkholm et al., 2018). Hence, the *D*_LCO/NO_ technique has previously been reported to yield largely similar exercise‐induced changes to those obtained by the $mF_{IO_{2}}$ technique (Coffman et al., 2016), while also exhibiting excellent test–retest reliability (Alves et al., 2022). Given that the supine position also leads to pulmonary capillary recruitment (Chang et al., 1992), while conceivably being easier to standardize than an acute exercise bout, it may be worthwhile to consider this as an alternative tool for assessing alveolar–capillary reserve using the *D*_LCO/NO_ technique. Expectedly, such changes would, however, be less pronounced than during exercise (Ross et al., 2020), but whether this affects the test–retest reliability of *D*_LCO/NO_ metrics remains to be determined.

Somewhat surprisingly, the test–retest reliability of *D*_LNO_ measured during exercise by the *D*_LCO/NO_ technique was recently reported to be better than in previous studies where measurements were obtained during normal upright rest (Alves et al., 2022). However, no studies have yet reported direct comparisons of the test–retest reliability of *D*_LNO_, D_LCO_, or various derived measures such as *D*_M_ and *V*_c_ during upright rest versus exercise versus the supine position. In theory, nitric oxide (NO) is a superior diffusion tracer compared to CO, because NO reacts with haemoglobin nearly 1500 times faster, and is consequently far less dependent on *V*_c_, therefore rendering it a ‘purer’ tracer of diffusion across the alveolar–capillary membrane. In contrast to D_LCO_, *D*_LNO_ is thus relatively unaffected by $F_{IO_{2}}$, ambient pressure or the presence of carboxyhaemoglobin (Zavorsky & van der Lee, 2017). In contrast to *D*_M_ and *V*_c_, *D*_LNO_ measures are furthermore based on fewer assumptions and mathematical derivations. Of all the *D*_LCO/NO_ metrics, *D*_LNO_ could potentially comprise the best parameter for assessing alveolar–capillary reserve during various conditions, such as exercise and the resting supine position.

In the present study, we investigated the between‐day test–retest reliability of *D*_LCO/NO_‐based measures of *D*_L_ in healthy volunteers during upright rest, and in two conditions: exercise and the resting supine position. We hypothesized that (1) exercise leads to more prominent changes in various *D*_LCO/NO_ metrics compared to the resting supine position, but with the same day‐to‐day reliability, and (2) *D*_LNO_ is more reliable during conditions than during upright rest, and furthermore yields superior reliability estimates compared with D_LCO_.

## 2 METHODS

### 2.1 Participants

Twenty healthy men and women were recruited to participate in the study, from July 2021 to September 2021. The participants gave both verbal and written consent to participate in the study, which was approved by the Ethical Committee for the Capital Region of Denmark (H‐20052659) and conducted in accordance with the *Declaration of Helsinki*. The study was registered at www.clinicaltrials.gov (NCT04963504). The participants were physically active, non‐smokers with normal ventilatory and diffusing capacity.

### 2.2 Experimental set‐up

#### 2.2.1 Study design overview

**FIGURE 1:**
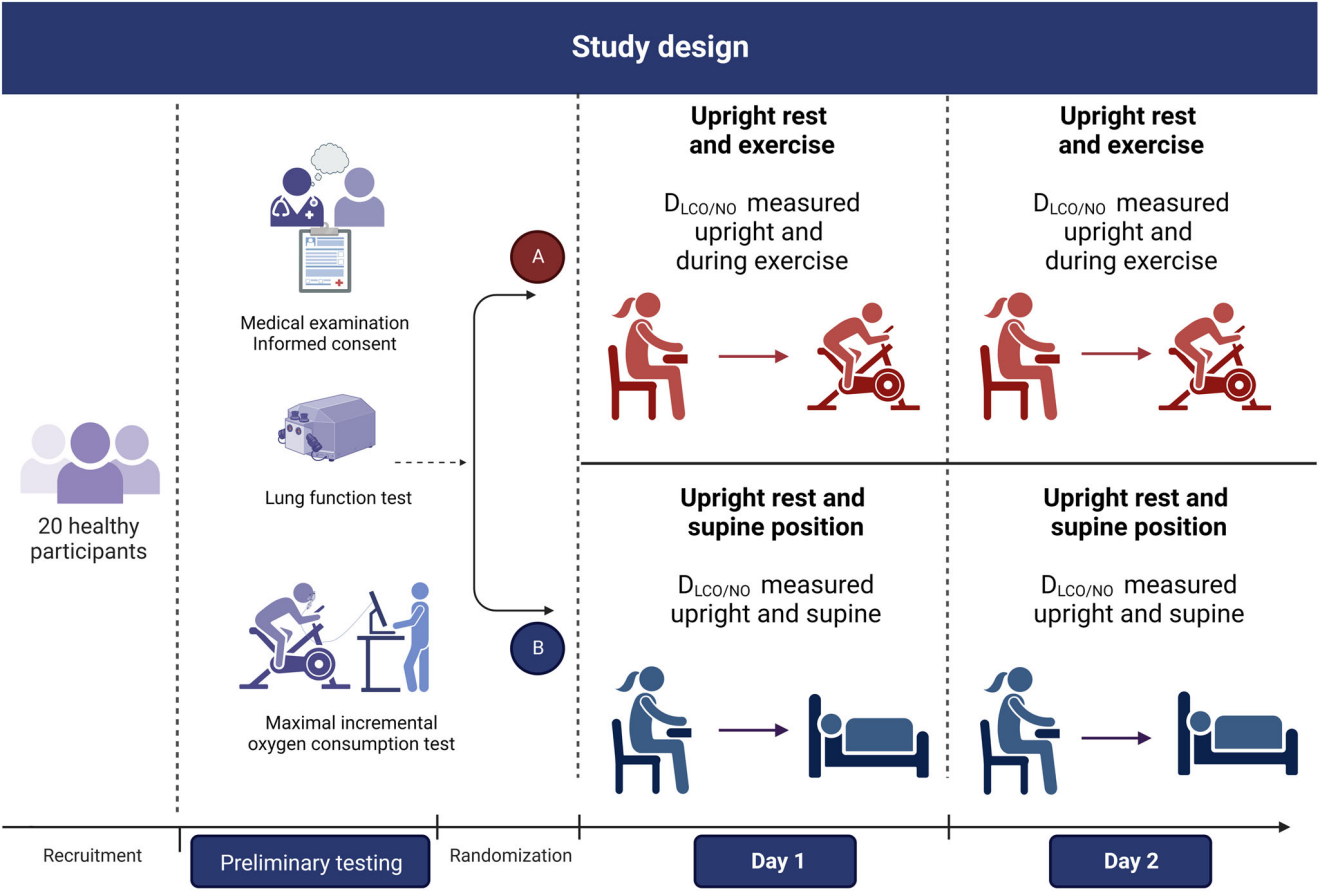
Overview of the study design. After preliminary testing, participants were randomized into two groups, and performed *D*_LCO/NO_ measurements in the upright sitting position followed by either a measurement during exercise (Group A) or in the resting supine position (Group B). An identical visit was repeated within 1–7 days. *D*_LCO/NO_, pulmonary diffusing capacity for carbon monoxide and nitric oxide

An overview of the study design is presented in Figure 1. Participants underwent preliminary testing, including a full pulmonary function test and a maximal oxygen consumption test (${\dot{V}}_{O_{2}\max}$), and were then randomized into two groups with two visits, here designated Day 1 and Day 2 of the study. In the first group (Group A), *D*_LCO/NO_ was measured at rest in the sitting position followed by measurements during exercise on a bicycle ergometer. In the second group (Group B), *D*_LCO/NO_ was measured at rest in the sitting position followed by measurements in the resting supine position. For both groups, the visit was repeated within 1–7 days.

#### 2.2.2 Preliminary testing

Participants underwent a medical health interview and examination, anthropometric measurements, and a full standardized lung function test, including dynamic spirometry, whole‐body plethysmography and single‐breath diffusing capacity to carbon monoxide (Jaeger Masterscreen PFT pro, CareFusion, Höchberg, Germany), which were conducted in accordance with the current consensus guidelines (MacIntyre et al., 2005; Miller et al., 2005; Wanger et al., 2005). Based on established summary equations, the expected values of forced expiratory volume in 1 s (FEV_1_), forced vital capacity (FVC), total lung capacity (TLC), residual volume (RV) and haemoglobin corrected *D*_LCO_ (*D*_LCOc_) according to age and anthropometry were calculated (Cotes et al., 1993; Quanjer et al., 1993). Lastly, the participants completed a ${\dot{V}}_{O_{2}\max}$ test on a bicycle ergometer (E839, Monark Exercise AB, Vansbro, Sweden). Initial workload was set to 70 W for a 5‐min warm‐up followed by a 15 W increase every minute until exhaustion. Oxygen consumption was measured breath‐by‐breath using a Quark gas analyser (Cosmed Quark CPET System, Cosmed Srl, Rome, Italy). ${\dot{V}}_{O_{2}\max}$ was considered achieved if two out of three of the following criteria were fulfilled: (1) an increase in oxygen consumption <100 mL/min with an increase in power output; (2) respiratory exchange ratio >1.1; and (3) reaching ±10 beats per minute of age‐predicted maximal heart rate (HR) (Bassett & Howley, 2000). HR was measured in beats per minute with a Garmin HR chest band compatible with the Cosmed system (Garmin International Inc., Olathe, KS, US).

#### 2.2.3 Experimental procedures

*D*_LCO/NO_ was determined using the single‐breath manoeuvre (Jaeger Masterscreen PFT pro, CareFusion) and in accordance with established quality criteria for clinical use, described elsewhere (Munkholm et al., 2018). Briefly, the participant was sitting and equipped with a nose clip. After an automatic resetting of the device the participant began normal tidal respiration through a mouthpiece (SpiroBac; Henrotech, Aartselaar, Belgium) (dead space 56 ml, resistance to flow at 12 L s^−1^ 0.9 cmH_2_O) connected to a pneumotach. The participant was instructed to exhale to residual volume followed by a rapid full inspiration by which a valve opened, allowing the participant to inhale the test gases (0.28% CO, 9.3% He, 20.9% O_2_ and 69.52% N_2_), which was mixed with 800 ppm NO/N_2_ in an inspiratory bag just before inhalation. After the inspiration, a breath‐hold of 5 s was performed, and participants were then guided to do a quick exhalation. The measurement was repeated after a 4‐min washout period and additional manoeuvres were performed until two manoeuvres fulfilled the repeatability criteria (Munkholm et al., 2018; Zavorsky et al., 2017). Acceptable repeatability was achieved if the two measurements varied <5.8 mmol/(min kPa) for *D*_LNO_, <1 mmol/(min kPa) for D_LCO_, <11 mmol/(min kPa) for *D*_M_ and <10 mL for *V*_c_ (Zavorsky et al., 2017). The standing height (nearest 1 mm) and weight (nearest 100 g) were obtained at baseline for each participant and used throughout the study. Haemoglobin was measured from capillary blood (HemoCue, Denmark) on each study day.

#### 2.2.4 Exercise *D*_LCO/NO_ trial

Following the *D*_LCO/NO_ measurement in the sitting upright resting condition, Group A performed the measurements during exercise. The participant was placed on a bicycle ergometer (identical to that used during the preliminary testing; E839, Monark Exercise) and began exercising at an intensity corresponding to 90% of the ventilatory threshold. This is a targeted workload similar to that of a recent study also assessing *D*_LNO_ during exercise (Alves et al., 2022). The individual ventilatory threshold was determined using the V‐slope method, expressing the linearity of carbon dioxide release as function of ${\dot{V}}_{O_{2}}$ (Beaver et al., 1986).

The *D*_LCO/NO_ measurements were made after 2 min, to ensure steady state. The measurement was repeated after a 4‐min washout period, until two measurements fulfilled the repeatability criteria described above. Between measurements the participant continued to exercise at an intensity that was pragmatically set at a workload that corresponded to 70% of the ventilatory threshold.

#### 2.2.5 Supine *D*_LCO/NO_ trial

For Group B, the sitting *D*_LCO/NO_ measurements were followed by a measurement in the supine position. The participants were placed in the supine position for 15 min before obtaining the *D*_LCO/NO_ measurement. Likewise, the measurements were repeated after a 4‐min washout period. The measurements were accepted if all repeatability criteria, described above, were met.

#### 2.2.6 Calculation of *D*_M_, *V*_c_ and alveolar volume

*D*_M_ and *V*_c_ were calculated as follows:

$$D_{M}=\frac{\frac{1}{\alpha }-\frac{1}{k}}{\frac{1}{D_{\mathrm{LNO}}}-\frac{1}{k\;\times D_{\mathrm{LCO}}}}$$

$$V_{c}=\frac{\left(\frac{1}{\theta_{\mathrm{CO}}}\right)\left(1-\frac{\alpha }{k}\right)}{\frac{1}{D_{\mathrm{LCO}}}-\frac{\alpha }{D_{\mathrm{LNO}}}}$$

where $\frac{1}{\theta_{\mathrm{CO}}}$ was calculated as previously described (Munkholm et al., 2018), α was set to 1.97, and *k* was calculated as: θ_NO_/θ_CO_, of which θ_NO_ was set at a finite value of 13.46 mL NO mL blood^−1^ min^−1^ kPa^−1^ (4.5 mL NO mL blood^−1^ min^−1^ mmHg^−1^) (Zavorsky et al., 2017). Five‐second alveolar volume (*V*_A_) was estimated as described elsewhere (Munkholm et al., 2018). Because *D*_LCO_ and by consequence its derived metrics may be affected notably by alveolar oxygen tension ($P_{AO_{2}}$), which varies through between‐day and between‐manoeuvre variations in $F_{IO_{2}}$, ambient pressure and alveolar ventilation, we also calculated O_2_‐adjusted *D*_LCOc_ (*D*_LCOcAdj_), *D*_M_ (*D*_MAdj_) *V*_c_ (*V*_cAdj_) and *V*_A_ (*V*_AAdj_) using the same approach as previously (Munkholm et al., 2018).

### 2.3 Statistical analysis

All statistical analyses were performed using R statistical software version 4.1.1 (R Foundation for Statistical Computing, Vienna, Austria) within RStudio (version 1.4.1717), and a two‐tailed *P* < 0.05 was considered statistically significant. Data are presented as mean (standard deviation (SD)) and mean difference (95% confidence interval (95% CI): lower limit (LL), upper limit (UL)), unless otherwise stated. Multiple Student's paired *t*‐tests were used to test between‐day differences of *D*_LCO/NO_ metrics. To account for multiple comparisons, *P*‐values were Holm–Bonferroni corrected, and an adjusted two‐tailed *P* < 0.05 was considered statistically significant.

We assessed the test–retest reliability of *D*_LCO/NO_ metrics during upright rest, exercise and in the supine position. Reliability considers the magnitude of random error due to the inherent variability in the measured variable (Bartlett & Frost, 2008). Firstly, interval consistency was assessed by paired *t*‐tests between identical conditions on separate study days. Furthermore, *absolute reliability*, which is quantified in the same units as the measurement, was evaluated by Bland–Altman plots and limits of agreement (LOA) computed from standard formulae (Bland & Altman, 1986), and the closely related smallest real difference (SRD), an estimate of how much difference there will be between two measurements in 95% of the occasions (Vaz et al., 2013), was also determined. SRD was obtained by a one‐way analysis of variance (ANOVA) to assess the standard deviation within the participants (SD_w_), and SRD was subsequently estimated with the following formula:

$$\begin{array}{ccc} \mathrm{SRD} & = & (t-\mathrm{quantile}\;\mathrm{with}\;\mathrm{the}\;\mathrm{appropriate}\;\mathrm{number}\\ & & \;\mathrm{of}\;\mathrm{degrees}\;\mathrm{of}\;\mathrm{freedom})\times \sqrt{2\times S{D_{w}}^{2}} \end{array}$$

SRD was specifically used to compare the reliability of a given *D*_LCO/NO_ metric between different conditions (upright rest, exercise and supine position). To further enable comparisons of the reliability of different *D*_LCO/NO_ metrics within each condition, the coefficient of variation (CV) was used as a relative reliability estimate. CV estimates as a percentage how much of the variance between the two study days was being caused by measurement error:

$$\mathrm{CV}=\frac{SD_{w}}{\mathrm{mean}\;\mathrm{of}\;\mathrm{measurements}\;}\times 100$$

Based on the distribution of the estimates of mean and residual variance from a linear mixed model, we simulated the distribution of the CV to obtain 95% confidence intervals for the CV.

The intra‐class correlation coefficient (ICC), based on a two‐way mixed effect model with absolute agreement and multiple measurements (ICC_3,_ *_k_*), was used as an additional relative reliability estimate.

Both absolute and relative reliability refer to either the repeatability or the reproducibility of a measurement, of which the former refers to the ability of a method to obtain the same results under identical conditions, whereas the latter reflects the ability to obtain the same results under changing conditions (Bartlett & Frost, 2008). Even though our test–retest assessments were conducted on separate days, the experimental conditions were entirely identical, in the same room, at the same time of day, with the same equipment, and the same operators. Furthermore, none of the *D*_LCO/NO_ metrics were likely to exhibit any physiological changes within the ≤7‐day time span between experiments. Thus, repeatability rather than reproducibility was considered in the present study. All the used statistical codes for R are provided online (see Supporting information, Supplemental File 1).

## 3 RESULTS

All subjects completed and tolerated the design of the study. The baseline characteristics of the participants are outlined in Table 1. A total of 80 *D*_LCO/NO_ measurements were obtained in the study; in 75 cases, the measurement was based on two manoeuvres. In the remaining five, one at rest, two during exercise and two in the supine position, a third manoeuvre was necessary to fulfil the repeatability criteria. Results of the *D*_LCO/NO_ measurements on each study day and during each condition are presented in Table 2. The average time between measurements was 2.5 (1.5) days.

**TABLE 1:** Baseline characteristics

Characteristic	Value
Sex (F/M)	10/10
Age (years)	24.8 ± 1.5
Height (cm)	175.2 ± 8.3
Weight (kg)	72.6 ± 13
FEV_1_ (L)	4.4 ± 0.8
FEV_1_ (% of predicted)	105.0 ± 10.5
FVC (L)	5.4 ± 1.0
FVC (% of predicted)	109.2 ± 10.1
FEV_1_/FVC ratio	0.814 ± 0.1
FEV_1_/FVC ratio (% of predicted)	95.7 ± 7.6
TLC (L)	6.2 ± 1.2
TLC (% of predicted)	92.7 ± 23.0
RV (L)	1.7 ± 0.4
RV (% of predicted)	104.6 ± 20.1
*D*_LCOc_ (mmol/(min kPa))	10.4 ± 2.1
*D*_LCOc_ (% of predicted)	92.7 ± 9.8
${\dot{V}}_{O_{2}\max}$ (mL/min)	3378.7 ± 870.1
${\dot{V}}_{O_{2}\max}$ (mL/min/kg)	46.1 ± 6.3

Abbreviations: *D*_LCOc_, pulmonary diffusing capacity for carbon monoxide corrected for haemoglobin; FEV_1_, forced expiratory volume in 1 s; FVC, forced vital capacity; RV, residual volume; TLC, total lung capacity; ${\dot{V}}_{O_{2}\max}$, maximal oxygen consumption.

**TABLE 2:** Physiological responses at rest, during exercise and postural changes

	Group A (*n* = 11)						Group B (*n* = 9)					
	Upright rest			Exercise			Upright rest			Supine position		
	Day 1	Day 2	*P*	Day 1	Day 2	*P*	Day 1	Day 2	*P*	Day 1	Day 2	*P*
*D*_LNO_ (mmol/(min kPa))	44.9 (8.9)	46.4 (8.9)	0.577	57.7 (10.3)	58.1 (10.8)	1.000	40.1 (7.2)	41.1 (8.2)	1.000	43.1 (8.4)	42.8 (8.6)	1.000
*D*_LCOc_ (mmol/(min kPa))	9.79 (1.9)	10.1 (2.0)	0.420	13.1 (2.4)	13.5 (2.8)	0.730	9.0 (1.7)	8.9 (1.8)	1.000	10.6 (2.2)	10.6 (2.2)	1.000
*D*_M_ (mmol/(min kPa))	22.8 (4.5)	23.1 (4.5)	0.577	29.3 (5.2)	29.5 (5.5)	1.000	20.7 (3.7)	21.0 (4.2)	1.000	21.9 (4.2)	21.7 (4.4)	1.000
*V*_c_ (mL)	76.5 (15.7)	77.6 (15.7)	0.551	89.9 (17.1)	92.0 (19.3)	0.672	71.6 (14.5)	69.1 (13.8)	0.640	94.2 (20.2)	91.6 (20.1)	1.000
*D*_LNO_/*D*_LCOc_ ratio	4.6 (0.2)	4.6 (0.2)	1.000	4.4 (0.2)	4.3 (0.2)	1.000	4.5 (0.2)	4.6 (0.1)	0.203	4.1 (0.3)	4.1 (0.2)	1.000
*V*_A_ (L)	6.00 (0.99)	6.06 (1.00)	0.594	6.73 (1.09)	6.70 (1.03)	1.000	5.35 (0.86)	5.39 (0.90)	1.000	5.10 (0.87)	5.08 (0.85)	1.000
Breath‐hold time (s)	6.2 (0.4)	6.0 (0.2)	0.383	5.9 (0.2)	5.8 (0.2)	0.191	6.1 (0.4)	5.9 (0.3)	1.000	6.0 (0.3)	6.0 (0.3)	1.000

Multiple paired *t*‐tests were used to test between‐day differences of *D*_LCO/NO_ metrics. *P*‐values are Holm–Bonferroni adjusted and a two‐tailed *P*‐value of *P* < 0.05 is considered statistically significant. Data are presented as mean (SD).

Abbreviations: CV, coefficient of variation; *D*_LCOc_, pulmonary diffusing capacity for carbon monoxide corrected for hemoglobin; *D*_LNO_, pulmonary diffusing capacity for nitric oxide; *D*_M_, membrane diffusing capacity; ICC, intraclass correlation coefficient; SRD, smallest real difference; *V*_A_, alveolar volume; *V*_c_, pulmonary capillary blood volume.

### 3.1 Exercise

**FIGURE 2:**
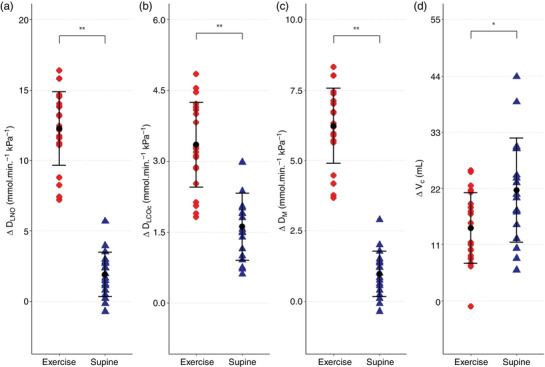
The physiological responses during exercise and postural changes. The figure displays the physiological change for *D*_LNO_ (a), *D*_LCOc_ (b), *D*_M_ (c), and *V*_c_ (d) from upright rest to exercise (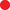) and from upright rest to the supine position (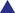). Black mark and line show the mean ± SD. **P < 0.0001, *P = 0.049. The repeated measurements during each condition are combined in the plot (Exercise: *n* = 11 × 2, Supine: *n* = 9 × 2). *D*_LCOc_, pulmonary diffusing capacity for carbon monoxide corrected for haemoglobin; *D*_LNO_, pulmonary diffusing capacity for nitric oxide; *D*_M_, membrane diffusing capacity; *V*_c_, pulmonary capillary blood volume

The mean workload during ergometer cycling was 203 (42) W, equivalent to 66 (4)% of the maximal workload achieved during the previous ${\dot{V}}_{O_{2}\max}$ test. *D*_LNO_, *D*_LCOc_, *D*_M_ and *V*_c_ all increased from upright rest to exercise (Δ change) on both study days (Figure 2). *V*_A_ increased in response to exercise (Day 1: Δ0.74 (0.2) L (*P* < 0.0001); Day 2: Δ0.65 (0.2) L (*P* < 0.0001)), while *D*_LNO_/*D*_LCOc_ ratio decreased (Day 1: Δ0.176 (0.2) (*P* = 0.008); Day 2: Δ0.261 (0.1) (*P* < 0.0001)).

### 3.2 Supine position

On both study days, *D*_LNO_, *D*_LCOc_, *D*_M_, and *V*_c_ all increased in response to a postural change from the upright rest to the supine position (Figure 2). The *V*_A_ decreased from upright rest to the supine position (Day 1: Δ0.263 (0.3) L (*P* = 0.014); Day 2: Δ0.308 (0.2) L (*P* = 0.002)) in parallel with the *D*_LNO_/ *D*_LCOc_ ratio (Day 1: Δ0.439 (0.3) (*P* = 0.001); Day 2: Δ0.582 (0.1) (*P* < 0.0001)).

The increases from upright rest to the supine position for *D*_LNO_, *D*_LCOc_ and *D*_M_ were less pronounced compared to the increase from upright rest to exercise with mean differences of 10.4 (95% CI: 8.5, 12.2) mmol/(min kPa) for *D*_LNO_, 1.7 (95% CI: 1.0, 2.5) mmol/(min kPa) for *D*_LCOc_ and 5.3 (95% CI: 4.3, 6.29) mmol/(min kPa) for *D*_M_, all *P* < 0.001. In contrast, Δ*V*_c_ was greater in the upright rest to supine position than during upright rest to exercise with a mean difference between conditions of 7.4 (95% CI: 0.5, 14.8) mL (*P* = 0.049) (Figure 2).

### 3.3 Test–retest reliability

**FIGURE 3:**
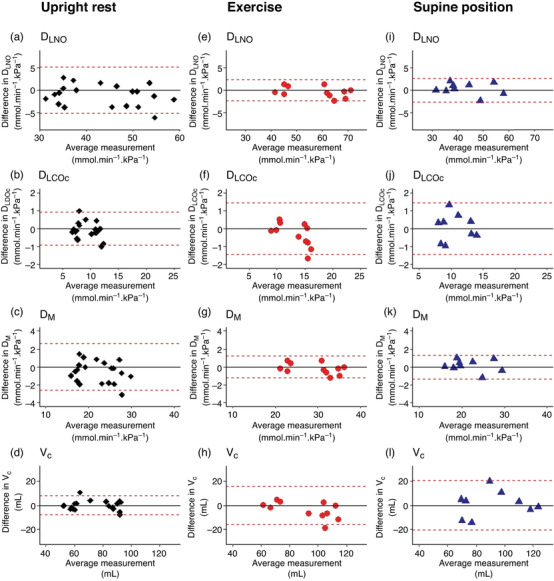
Between‐day test–retest reliability of *D*_LCO/NO_ metrics according to Bland–Altman plots. The dotted lines show upper and lower 95% limits of agreement (LOA). The between‐day reliability is shown for *D*_LNO_ (a, e, i), *D*_LCOc_ (b, f, j), *D*_M_ (c, g, k) and *V*_c_ (d, h, l) during upright rest (♦, *n* = 20), exercise (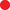, *n* = 11) and in the supine position (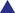, *n* = 9). *D*_LCOc_, pulmonary diffusing capacity for carbon monoxide corrected for haemoglobin; *D*_LNO_, pulmonary diffusing capacity for nitric oxide; *D*_M_, membrane diffusing capacity; *V*_c_, pulmonary capillary blood volume

In terms of internal consistency, there were no between‐day differences between *D*_LCO/NO_ metrics obtained in each condition (Table 2). Bland–Altman plots with LOA for *D*_LNO_, *D*_LCOc_, *D*_M_ and *V*_c_ are provided in Figure 3.

SRD, CV and ICC values for each condition are presented in Table 3. SRD for *D*_LNO_ was lower during exercise than during upright rest, which was not the case in the supine position; a similar pattern was evident for *D*_M_. SRD values for *D*_LCOc_ were similar in all conditions. As for *V*_c_, SRD was higher in the supine position than upright rest, but similar between the upright rest and exercise. SRD values for *V*_A_ were similar in all conditions (0.20 (95% CI: 0.16, 0.26), 0.27 (95% CI: 0.21, 0.37) and 0.26 (95% CI: 0.17, 0.49) for upright rest, exercise and supine position, respectively). During upright rest, CV was similar for all metrics apart from *V*_A_, in which it was notably low at 1.2% (95% CI: 1.0, 1.3), 1.3% (95% CI: 1.1, 1.4) and 1.6% (95% CI: 1.3, 1.8) for upright rest, exercise and supine position, respectively. During exercise and in the supine position, CV values for *D*_LNO_ and *D*_M_ were lower than for *D*_LCOc_ and *V*_c_. According to ICC, the reliability of all metrics was >0.90 in all conditions, except for *V*_c_. ICC for *V*_A_ was 1 (95% CI: 0.99, 1.00) in all conditions. Of note, O_2_‐adjustment of the *D*_LCOc_‐derived variables did not improve any of the reliability estimates (see Supporting information, Supplemental File 2). The *D*_LNO_/*D*_LCOc_ ratio showed a SRD value of 0.3 (95% CI: 0.3, 0.4), 0.4 (95% CI: 0.2, 0.8) and 0.6 (95% CI: 0.4, 1.0) during upright rest, exercise and in the supine position, respectively. It had a CV of 2.3 (95% CI: 2.0, 2.5)% during upright rest, 2.8 (95% CI:2.4, 3.2)% during exercise and 4.4 (95% CI: 3.6, 4.9)% in the supine position. ICC was 0.82 (95% CI: 0.62, 0.92) at upright rest, 0.83 (0.50, 0.94) during exercise and 0.62 (95% CI: 0.25, 0.89) in the supine position.

**TABLE 3:** Reliability of *D*_LCO/NO_ metrics

	Upright rest Group A + B (*n* = 20)			Exercise Group A (*n* = 11)			Supine position Group B (*n* = 9)		
	SRD (units)	CV (%)	ICC (fraction)	SRD (units)	CV (%)	ICC (fraction)	SRD (units)	CV (%)	ICC (fraction)
*D*_LNO_ (mmol/(min kPa))	5.4 (4.1, 7.5)	4.2 (3.7, 4.6)	0.98 (0.96, 0.99)	2.7 (2.0, 3.9)	1.5 (1.2, 1.7)	1.00 (0.99, 1.00)	3.0 (2.1, 4.8)	2.2 (1.8, 2.5)	0.99 (0.98, 1.00)
*D*_LCOc_ (mmol/(min kPa))	1.0 (0.7, 1.4)	3.4 (3.0, 3.8)	0.99 (0.97, 0.99)	1.6 (1.1, 2.8)	3.8 (3.2, 4.3)	0.99 (0.96, 1.00)	1.6 (1.2, 2.5)	4.8 (3.8, 5.4)	0.98 (0.92, 0.99)
*D*_M_ (mmol/(min kPa))	2.7 (2.1, 3.8)	4.2 (3.7, 4.6)	0.98 (0.96, 0.99)	1.4 (1.0, 2.0)	1.5 (1.2, 1.7)	1.00 (0.99, 1.00)	1.5 (1.1, 2.4)	2.2 (1.7, 2.5)	0.99 (0.98, 1.00)
*V*_c_ (mL)	8.4 (6.1, 12.7)	3.8 (3.3, 4.2)	0.98 (0.96, 0.99)	17.8 (11.7, 31.9)	6.1 (5.1, 6.9)	0.97 (0.90, 0.99)	24.0 (16.7, 38.6)	8.0 (6.4, 9.1)	0.94 (0.80, 0.98)
*V*_A_ (L)	0.20 (0.16, 0.26)	1.2 (1.0, 1.3)	1.00 (1.00, 1.00)	0.27 (0.21, 0.37)	1.3 (1.1, 1.4)	1.00 (0.99, 1.00)	0.26 (0.17, 0.49)	1.6 (1.3, 1.8)	1.00 (0.99, 1.00)

Data are presented with 95% CI (LL, UL). Abbreviations: CV, coefficient of variation; *D*_LCOc_, pulmonary diffusing capacity for carbon monoxide corrected for haemoglobin; *D*_LNO_, pulmonary diffusing capacity for nitric oxide; *D*_M_, membrane diffusing capacity; ICC, intraclass correlation coefficient; SRD, smallest real difference; *V*_A_, alveolar volume; *V*_c_, pulmonary capillary blood volume.

## 4 DISCUSSION

The present study is the first to assess and compare the reliability of *D*_LCO/NO_ metrics during exercise to the resting supine position. We found a slightly better reliability of *D*_LNO_ and *D*_M_, yielding lower CV during exercise than in the supine position. Furthermore, while the reliability measures of *D*_LNO_, *D*_LCOc_, *D*_M_ and *V*_c_ were similar during upright rest, only the reliability of *D*_LNO_ and *D*_M_ increased during exercise, so that they became superior to *D*_LCOc_ and *V*_c_, as indicated by lower CV values. The *D*_LNO_/*D*_LCOc_ ratio did not appear to be reliable in any condition.

The reported exercise‐induced changes in *D*_LNO_, *D*_LCOc_, *D*_M_ and *V*_c_ indicate that during submaximal exercise, blood is mobilized to the pulmonary capillaries to increase *V*_c_ with concomitant pulmonary capillary recruitment and distension, increasing the area of the alveolar–capillary interface available for gas exchange and thus *D*_M_. These changes largely agree with findings from previous studies on healthy volunteers at various moderate to vigorous exercise intensities, based on both the *D*_LCO/NO_ technique (Alves et al., 2022; Coffman et al., n.d.; Jorgenson et al., 2018) and the $mF_{IO_{2}}$ technique (Michaelchuk et al., 2019; Tedjasaputra et al., 2016).

The impact of the supine position on *D*_LCO/NO_ metrics has not previously been investigated. In a study using the $mF_{IO_{2}}$ technique on healthy volunteers, similar effects on D_LCO_, *D*_M_ and *V*_c_ have been reported (Chang et al., 1992). This is also supported by a previous study in which D_LCO_ was found to increase similarly by 20% in the supine compared to the sitting position with a decrease in baseline thoracic electrical impedance reflecting a larger central blood volume and thus *V*_c_ (Hanel et al., 1997). However, other studies have found no effects of the supine position on D_LCO_, *D*_M_ or *V*_c_ (Pistelli et al., 1991; Ross et al., 2020), and the cause of the discrepancy between the findings in these studies is not immediately apparent. According to our findings, the supine position evokes similar although less pronounced changes in the different *D*_LCO/NO_ metrics compared to exercise, apart from a reduction rather than an increase in *V*_A_. The latter probably reflects the well‐known reduction of lung volumes in the supine position, partly because the abdominal contents are moved cranially, due to shortening of the thorax in the apical‐to‐basal direction, and partly because the direction of the gravitational vector relative is changed, thus causing the thorax to be slightly compressed in the ventral‐to‐dorsal direction (Berg et al., 2022). The concomitant increase in *V*_c_, which exceeds that observed during exercise with only a relatively small increase in *D*_M_, may suggest that fewer capillaries are available for recruitment than during upright exercise, so that capillary distension becomes dominant. However, of all *D*_LCO/NO_ metrics, *V*_c_ yielded the poorest reliability, and these findings should therefore be interpreted with caution. Aside from *V*_c_, the supine position did provide reliable *D*_LCO/NO_ metrics and may thus provide useful surrogate indices of alveolar–capillary reserve in studies where measurement during exercise is not feasible.

In accordance with our working hypothesis and recent findings (Alves et al., 2022), exercise markedly increased the reliability of *D*_LNO_, and both during exercise and in the resting supine position, it was higher than that of *D*_LCOc_. However, it must be noted that our exact reliability estimates are not directly comparable to those from previous studies, either at rest (Desjardin et al., 2020; Murias & Zavorsky, 2007) or during exercise (Alves et al., 2022). This is notably because these previous studies used the standard formula of 1.96 √(2 SD_w_) to calculate SRD (Bland & Altman, 1986), corresponding to a constant of 2.77 SD_W_, but this presupposes that the sample size is sufficiently large (*n* > 500). Since this is rarely feasible in human‐experimental studies, we accounted for the small sample sizes in the present study and calculated an accurate constant based on a *t*‐distribution with the appropriate degrees of freedom. Therefore, the present study used a constant of 2.95, 3.14 and 3.26 for upright rest, exercise and the resting supine position, respectively. On this basis, our findings do confirm that better reliability may be achieved during exercise than during upright rest, both for *D*_LNO_ and for *D*_M_, but not for *D*_LCOc_ or *V*_c_. It must, however, be addressed that this applies only to young healthy volunteers. Furthermore, an important limitation in the present study is the absence of physiological monitoring during exercise to ensure the same metabolic load on both study days. Further studies with more extensive physiological monitoring to assess metabolic load are required to uncover whether exercise also elicits better reliability in patients with chronic lung diseases, where exertional dyspnoea and related symptoms may render breath‐hold during *D*_LCO/NO_ manoeuvres particularly more difficult than in the supine position.

The finding that the reliability of *D*_LNO_ is superior to *D*_LCOc_ supports the contention that NO is a better diffusion tracer compared to CO (Zavorsky & van der Lee, 2017). Considering the test–retest reliability alone, that is, between‐day repeatability, our findings suggests that of all the *D*_LCO/NO_ metrics, *D*_LNO_ or alternatively *D*_M_ during either exercise or in the resting supine position, should be the physiological outcome measure of choice when investigating alveolar–capillary reserve.

In the present study, we choose to report SRD, CV and ICC, since reporting only one of these would be insufficient due to the respective limitations of these reliability estimates. SRD is particularly important for clinical translation, because it is reported in the same units as the measurement itself, so a difference less than SRD noted in an individual is most likely caused by measurement error (Vaz et al., 2013). A major drawback of SRD is that it cannot readily be used to compare the reliability of different measures. CV was reported to have a dimensionless outcome for the comparison of *D*_LCO/NO_ metrics in each condition. This provides an assessment of the relationship between the standard deviation within the group, also annotated standard error of measurement (SEM), and the mean (Liu, 2012). CV should be interpreted with caution if the measure has means close to zero as it becomes inaccurate, because the CV then approaches infinity, regardless of SEM. This was, however, not an issue in the present study.

Reporting and interpreting ICC should also be done with caution. Typically, an ICC below 0.50 is considered to indicate poor, between 0.50 and 0.75 moderate, between 0.75 and 0.90 good, and greater than 0.90 excellent test–retest reliability (Koo & Li, 2016). This would classify the test–retest reliability of all *D*_LCO/NO_ metrics apart from *V*_c_ in the present study as excellent. However, this adds very little information to that provided by SRD and CV, and it must be kept in mind that ICC estimates are influenced substantially by the variation within and between groups (Lee et al., 2012). A very heterogeneous study population may generate high ICC values, whereas a homogeneous population with the same measurement error will generate lower ICC values in comparison. Thus, as a test–retest reliability estimate, ICC cannot stand alone.

In conclusion, the *D*_LCO/NO_ technique generally provided reliable metrics in young healthy volunteers, during upright rest, exercise and in the supine position. There was evidence of pulmonary capillary recruitment both during exercise and in the supine position. The reliability estimates of *D*_LNO_ and *D*_M_ were notably increased during exercise and may thus be particularly useful indices of the alveolar–capillary reserve. Before they are implemented as physiological outcomes in clinical studies, it must, however, be confirmed that similar reliability estimates can be obtained in various patient populations.

## AUTHOR CONTRIBUTIONS

Conception, funding and supervision: Ronan M. G. Berg. Design: Ulrik Winning Iepsen, Regitse H. Chistensen, Jann Mortensen, Ronan M. G. Berg. Data collection: Anna Christrup Madsen, Rie Skovly Thomsen, Iben E. Rasmussen, Milan Mohammad, Birgitte Hanel, Simon Jønck. Data analysis: Stine B. Nymand, Jacob Peter Hartmann, Lene Theil Skovgaard. Writing – original draft: Stine B. Nymand, Ronan M. G. Berg. Review and editing: Anna Christrup Madsen, Rie Skovly Thomsen, Stine B. Nymand, Jacob Peter Hartmann, Iben E. Rasmussen, Milan Mohammad, Lene Theil Skovgaard, Birgitte Hanel, Simon Jønck, Ulrik Winning Iepsen, Regitse H. Chistensen, Jann Mortensen, Ronan M. G. Berg. All authors have read and approved the final version of this manuscript and agree to be accountable for all aspects of the work in ensuring that questions related to the accuracy or integrity of any part of the work are appropriately investigated and resolved. All persons designated as authors qualify for authorship, and all those who qualify for authorship are listed.

## CONFLICT OF INTEREST

None.

## Supporting information

Statistical Summary Document

Supplemental File 1

Supplemental File 2

Supplemental File 3

## Data Availability

The data that support the findings of this study are provided in Supporting information, Supplemental File 3.
