# Correction to ‘Pulmonary diffusing capacity to nitric oxide and carbon monoxide during exercise and in the supine position: A test–retest reliability study’

**DOI:** 10.1113/EP092280

**Published:** 2024-09-23

Madsen, A. C., Thomsen, R. S., Nymand, S. B., Hartmann, J. P., Rasmussen, I. E., Mohammad, M., Skovgaard, L. T., Hanel, B., Jønck, S., Iepsen, U. W., Christensen, R. H., Mortensen, J. and Berg, R. M. G. (2023). Pulmonary diffusing capacity to nitric oxide and carbon monoxide during exercise and in the supine position: A test–retest reliability study. *Experimental Physiology*, 108(2), 307–317. https://doi.org/10.1113/EP090883

There are some errors relating to reported alveolar–capillary membrane diffusing capacity (*D*_M_) and pulmonary capillary blood volume (*V*_c_) values in the original article. The authors incorrectly assumed that the manufacturer of the equipment (Jaeger MasterScreen PFT pro; CareFusion, Höchberg, Germany) used for the single‐beath measurement of the pulmonary diffusing capacity to carbon monoxide (corrected for haemoglobin, *D*_LCOc_) and nitric oxide (*D*_LNO_) reported *D*_M_ and *V*_c_ according to the established standard equations outlined in the article (Munkholm et al., 2018; Zavorsky et al., 2017).

However, the authors have recently realised that the equipment reports *D*_M_ as *D*_LNO_ divided by a constant of 1.97 instead, which subsequently affects the *V*_c_ estimates. This necessitates changes to the Results, Discussion, Tables 2 and 3 and Figures 2 and 3. The overall conclusion of the article, that *D*_LNO_ appears to be the most reliable metric relating to alveolar–capillary reserve during both exercise and in the supine position, remains unchanged by these errors. However, at several points in the article, *D*_M_ is mentioned as an alternative with comparable reliability, and these statements are now unfounded.

**TABLE 2:** Physiological responses at rest, during exercise and postural changes.

	Group A (*n* = 11)						Group B (*n* = 9)					
	Upright rest			Exercise			Upright rest			Supine position		
	Day 1	Day 2	P*	Day 1	Day 2	P*	Day 1	Day 2	P*	Day 1	Day 2	P*
*D*_LNO_ (mmol/(min·kPa))	44.9 (8.9)	46.4 (8.9)	0.577	57.7 (10.3)	58.1 (10.8)	1.000	40.1 (7.2)	41.1 (8.2)	1.000	43.1 (8.4)	42.8 (8.6)	1.000
*D*_LCOc_ (mmol/(min·kPa))	9.79 (1.9)	10.1 (2.0)	0.420	13.1 (2.4)	13.5 (2.8)	0.730	9.0 (1.7)	8.9 (1.8)	1.000	10.6 (2.2)	10.6 (2.2)	1.000
*D*_M_ (mmol/(min·kPa))	41.6 (11.7)	41.9 (10.0)	1.000	50.4 (12.4)	48.4 (10.0)	1.000	34.8 (8.0)	37.4 (9.0)	1.000	33.2 (8.0)	33.3 (7.7)	1.000
*V*_c_ (mL)	67.8 (12.7)	71.0 (12.5)	1.000	93.9 (17.6)	99.8 (21.9)	1.000	68.1 (13.9)	63.9 (13.0)	1.000	88.3 (21.7)	84.8 (18.2)	1.000
*D*_LNO_/*D*_LCOc_ ratio	4.6 (0.2)	4.6 (0.2)	1.000	4.4 (0.2)	4.3 (0.2)	1.000	4.5 (0.2)	4.6 (0.1)	0.203	4.1 (0.3)	4.1 (0.2)	1.000
V_A_ (L)	6.00 (0.99)	6.06 (1.00)	0.594	6.73 (1.09)	6.70 (1.03)	1.000	5.35 (0.86)	5.39 (0.90)	1.000	5.10 (0.87)	5.08 (0.85)	1.000
Breath‐hold time (s)	6.2 (0.4)	6.0 (0.2)	0.383	5.9 (0.2)	5.8 (0.2)	0.191	6.1 (0.4)	5.9 (0.3)	1.000	6.0 (0.3)	6.0 (0.3)	1.000

Multiple paired *t*‐tests were used to test between‐day differences of *D*_LCO/NO_ metrics. ^*^ *P*‐values are Holm–Bonferroni adjusted and a two‐tailed *P*‐value of *P* < 0.05 is considered statistically significant. Data are presented as mean (SD).

Abbreviations: CV, coefficient of variation; *D*_LCOc_, pulmonary diffusing capacity for carbon monoxide corrected for haemoglobin; *D*_LNO_, pulmonary diffusing capacity for nitric oxide; *D*_M_, membrane diffusing capacity; ICC, intraclass correlation coefficient; SRD, smallest real difference; *V*_A_, alveolar volume; *V*_c_, pulmonary capillary blood volume.

**TABLE 3:** Reliability of *D*_LCO/NO_ metrics.

	Upright rest Group A + B (*n* = 20)			Exercise Group A (*n* = 11)			Supine position Group B (*n* = 9)		
	SRD (units)	CV (%)	ICC (fraction)	SRD (units)	CV (%)	ICC (fraction)	SRD (units)	CV (%)	ICC (fraction)
*D*_LNO_ (mmol/(min·kPa))	5.4 (4.1, 7.5)	4.2 (3.7, 4.6)	0.98 (0.96, 0.99)	2.7 (2.0, 3.9)	1.5 (1.2, 1.7)	1.00 (0.99, 1.00)	3.0 (2.1, 4.8)	2.2 (1.8, 2.5)	0.99 (0.98, 1.00)
*D*_LCOc_ (mmol/(min·kPa))	1.0 (0.7, 1.4)	3.4 (3.0, 3.8)	0.99 (0.97, 0.99)	1.6 (1.1, 2.8)	3.8 (3.2, 4.3)	0.99 (0.96, 1.00)	1.6 (1.2, 2.5)	4.8 (3.8, 5.4)	0.98 (0.92, 0.99)
*D*_M_ (mmol/(min·kPa))	9.5 (7.6, 12.3)	8.1 (5.6, 11.0)	0.95 (0.89, 0.98)	10.3 (7.4, 15.7)	6.4 (3.8, 9.6)	0.96 (0.88, 0.99)	4.7 (3.3, 7.5)	4.2 (2.4, 6.6)	0.98 (0.94, 0.99)
*V*_c_ (mL)	16.2 (12.8, 21.5)	7.9 (5.6, 10.6)	0.90 (0.78, 0.95)	28.3 (19.7, 45.9)	9.0 (5.4, 13.4)	0.90 (0.71, 0.97)	30.5 (22.0, 46.3)	10.4 (5.8, 16.2)	0.88 (0.58, 0.56)
*V*_A_ (L)	0.20 (0.16, 0.26)	1.2 (1.0, 1.3)	1.00 (1.00, 1.00)	0.27 (0.21, 0.37)	1.3 (1.1, 1.4)	1.00 (0.99, 1.00)	0.26 (0.17, 0.49)	1.6 (1.3, 1.8)	1.00 (0.99, 1.00)

Data are presented with 95% CI (LL, UL).

Abbreviations: CV, coefficient of variation; *D*_LCOc_, pulmonary diffusing capacity for carbon monoxide corrected for haemoglobin; *D*_LNO_, pulmonary diffusing capacity for nitric oxide; *D*_M_, membrane diffusing capacity; ICC, intraclass correlation coefficient; SRD, smallest real difference; *V*_A_, alveolar volume; *V*_c_, pulmonary capillary blood volume.

**FIGURE 2:**
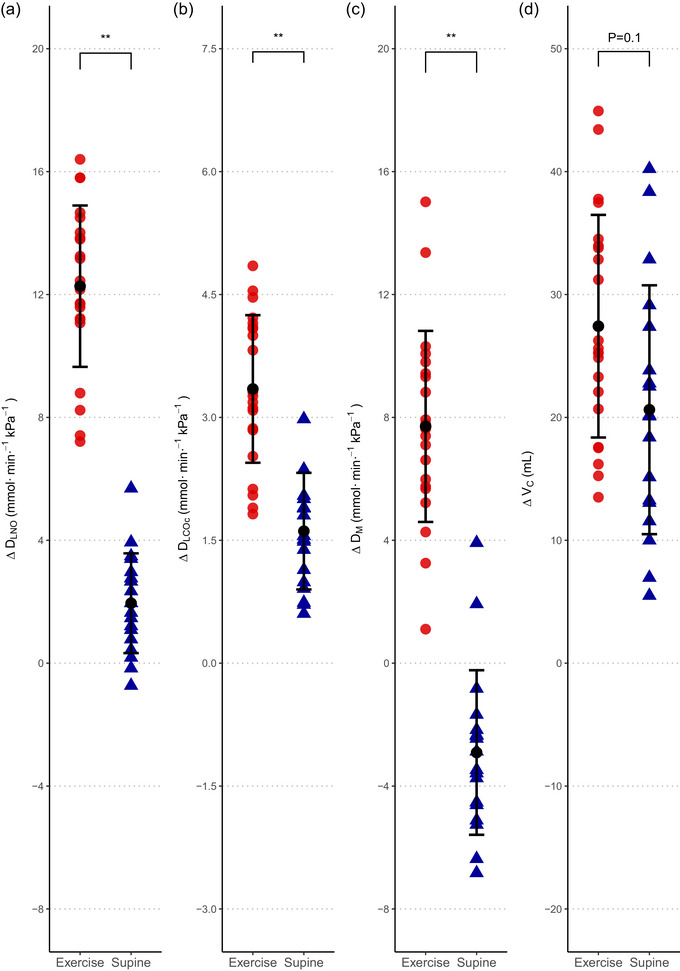
The physiological responses during exercise and postural changes. The figure displays the physiological change for (a) *D*_LNO_, (b) *D*_LCOc_, (c) *D*_M_ and (d) V_C_ from upright rest to exercise (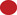) and from upright rest to the supine position (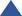). Black mark and line show the mean ± SD, ^**^ *P* < 0.0001. The repeated measurements during each condition are combined in the plot (Exercise: *n* = 11 × 2, Supine: *n* = 9 × 2). *D*_LCOc_, pulmonary diffusing capacity for carbon monoxide corrected for haemoglobin; *D*_LNO_, pulmonary diffusing capacity for nitric oxide; *D*_M_, membrane diffusing capacity; *V*_c_, pulmonary capillary blood volume.

**FIGURE 3:**
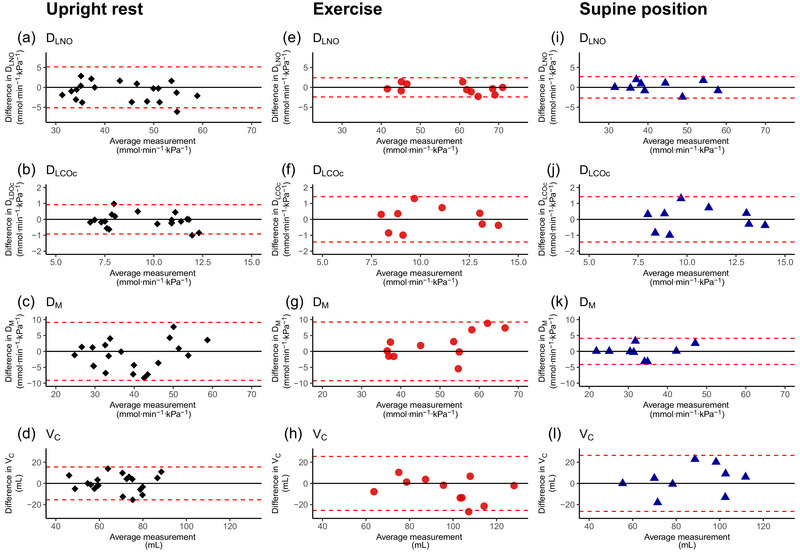
Between‐day test–retest reliability of *D*_LCO/NO_ metrics according to Bland–Altman plots. The dashed lines show upper and lower 95% limits of agreement (LOA). The between day reliability is shown for *D*_LNO_ (a, e, i), *D*_LCOc_ (b, f, j), *D*_M_ (c, g, k) and *V*_c_ (d, h, l) during upright rest (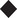, *n* = 20), exercise (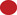, *n* = 11) and in the supine position (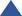, *n* = 9). *D*_LCOc_, pulmonary diffusing capacity for carbon monoxide corrected for haemoglobin; *D*_LNO_, pulmonary diffusing capacity for nitric oxide; *D*_M_, membrane diffusing capacity; *V*_c_, pulmonary capillary blood volume.

Corrected text, figures and tables can be found below:

## Results, Section 3.2 Supine position

- *D*_M_ decreases in response to a postural change from the upright rest to the supine position.
- Δ*D*_M_ is 10.6 (95% CI: 8.7, 12.5) mmol/(min kPa) greater from upright rest to exercise than from upright to the supine position (*P* < 0.001).
- Δ*V*_c_ from the upright to the supine position is similar to that from upright rest to exercise, with a mean difference between the Δ*V*_c_ in the two conditions of 6.8 (95% CI: −1.5, 15.1) mL (*P* = 0.104).

## Results, Section 3.4 Test–retest reliability

- SRD for *D*_M_ is similar during exercise and upright rest, and tends to be lower in the supine position.
- SRD for *V*_c_ is similar in the supine position and during exercise higher, and in both cases higher than at upright rest.
- During upright rest, *D*_M_ and *V*_c_ provide the highest CV values.
- During exercise and in the supine position, *D*_LNO_ has a lower CV than *D*_LCOc_, *D*_M_ and *V*_c_.

## Discussion

- Paragraph 1: Furthermore, while the reliability of *D*_LNO_, *D*_LCOc_, *D*_M_ and *V*_c_ were similar during upright rest, only the reliability of *D*_LNO_ increased during exercise, so that it was superior to that of *D*_LCOc_, *D*_M_ and *V*_c_ during the conditions, as indicated by lower CV values.
- Paragraph 4: Our findings do confirm that better reliability may be achieved during exercise than during upright rest for *D*_LNO_, but not for *D*_LCOc_, *D*_M_ or *V*_c_.
- Paragraph 5: Considering test–retest reliability alone, that is, between‐day repeatability, our findings suggests that of all the *D*_LCO/NO_ metrics, *D*_LNO_ during either exercise or in the resting supine position should be the physiological outcome measure of choice when investigating alveolar–capillary reserve.
- Paragraph 8: The reliability of *D*_LNO_ notably increased during exercise and may thus be a particularly useful index of the alveolar–capillary reserve.

Finally, this article corrects the name of author Regitse H. Christensen, who was incorrectly listed as Regitse H. Chistensen.

We apologise for these errors.
