# Supplementary material for: Pulmonary diffusing capacity to nitric oxide and carbon monoxide during exercise and in the supine position: a test–retest reliability study
Source: Exp Physiol. 2023 Jan 9;108(2):307–17. doi: 10.1113/EP090883 (PMC10103891; doi:10.1113/EP090883)
Supplement: Supplementary file 2 — Supplemental File 1 [file EPH-108-307-s004.pdf]

Online Supplemental File 1

```
# A script for all the analysis of Single-breath pulmonary diffusing capacity to  nitric oxide and carbon monoxide during
# acute exercise and in the supine position: a test-retest reliability study

# Created by: Stine Buus Nymand (September 2022)


# The codes are in the following order:

# 1) Repeatability coefficient / Smallest real difference

# 2) Coefficient of variance (CV)

# 3) Intra class correlation coefficient (ICC)

# 4) Bland Altman Plots


# We have chosen to use the metric DLNO during exercise as an example.

# -------------------------------------------------------------------------------------------------------------------------------

# set working director

d <- read.csv2("data.csv")

# 1) Repeatability coefficient / Smallest real difference


# Subset of rest to exercise (Group A)

names(d) [1] <- "ID"

exercise <- subset(d, Group_name == "Exercise")


# Subscript of days

exercise$Intervention <- NULL

exercise$Group_day <- NULL

exercise$Group_name <- NULL

d1 <-subset(exercise, Day=="1")

d2 <- subset(exercise, Day=="2")


# New data set with the squared differences - to be used later

diff <- data.frame((d1-d2)^2)
```

```
# Performing an ANOVA to calculate SDw
# Example with DLNOcondition
anova<-aov(DLNOcondition~factor(ID),data=exercise)
summary(anova)
StddevW<-sqrt(0.73) #Insert Mean sq. Residuals from summary anova

#RC or SRD is then calculated:
round(qt(0.975, 11-1)*sqrt(2)*StddevW,3)                    #Use correct df. We used 11

# To get 95% CI:
fit=glm(DLNOint~1 ,family=Gamma(link="log"),data=diff)  #Use the correct variable, and choose the data set diff squared
qt(0.975, 11-1)*sqrt(exp(coef(fit))) #insert correct df. We have used 11
qt(0.975, 11-1)*sqrt(exp(confint(fit))) #insert correct df. We have used 11.

# -----------------------------------------------------------------------------------------------------------------------------

# 2) Coefficient of variance (CV)
# We use the same model as used to calculate StddevW from the ANOVA.
#Examble with DLNOint (Group A: Exercise)
StddevW / mean(exercise$DLNOint) *100                       #Insert variable on x

# 95% CI:
#install.packages("nlme")
library("nlme")
model=lme(DLNOcondition~1, data=exercise, random=~1 | factor(ID))          # Insert variable on x
summary(model)
intervals(model)

antal=11                                                   #Insert correct df
```

```
sigma=   0.8524166                      #residual stdev
  omega=  11.05461                      #intercept stdev
  my=  57.92341                         #fixed effect intercept value
  sigma2=sigma^2
omega2=omega^2
varians=omega2/antal+sigma2/(2*antal)
spred=sqrt(varians)
s2=sigma2*rchisq(1:100000, df=11)/antal                          #insert df
ystreg=rnorm(1:100000,mean=my,sd=spred)
cv=sqrt(s2)/ystreg
round(summary(cv)*100,1)
round(quantile(cv,probs=c(0.025,0.25,0.5,0.75,0.975))*100,1)

# ------------------------------------------------------------------------------------------------------------------------------

# 3) Intra class correlation coefficient (ICC)

# First, we'll make a data set only for the specific variable for day 1 and day 2
ICC_DLNO_EXERCISE <- data.frame(d1$DLNOcondition, d2$DLNOcondition) #insert the correct variable on x1 and x2
head(ICC_DLNO_EXERCISE)
library(psych)
ICC(ICC_DLNO_EXERCISE)
#Choose the correct ICC. You can see; Koo & Li: A Guideline of Selecting and Reporting ICC...

# ------------------------------------------------------------------------------------------------------------------------------
# 4) Bland Altman Plot
# This Bland Altman Plot is made for repeated measurements
# First make a subset for days
d1 <- subset(exercise, Day=="1")
d2 <- subset(exercise, Day=="2")
```

```
df <- data.frame(Day1=d1$DLNOcondition, Day2=d2$DLNOcondition)
df$avg <- rowMeans(df)                    # avg of means
df$diff <- df$Day1-df$Day2                # The difference between the repeated measures
df$sq <- df$diff^2                        # Squared differences
RC <- 2* sqrt(sum(df$sq)/11)              # Remember to change df

#install.packages("ggplot2")
library(ggplot2)
a <- ggplot(df, aes(x = avg, y = diff)) +
  geom_point(size=2) +
  geom_hline(yintercept = 0) +
  geom_hline(yintercept = -RC, color = "red", linetype="dashed") +
  geom_hline(yintercept = RC, color = "red", linetype="dashed") +
  ggtitle("Bland Altman Plot- DLNO exercise") +
  ylab("Diffrence") +
  xlab("Average meassure")
a

# ----------------------------------------------------------------------------------------------------------------------------
# Calculation of difference between days
# Example with DLNO
#install.packages("nlme")
library("nlme")
# DLNO

d$diffDLNO <- d$DLNOsitting-d$DLNOcondition
model1=lme(diffDLNO~Intervention+factor(Day), data=d, random=~1 | factor(ID))
summary(model1)
intervals(model1)
```
