# Supplementary material for: Pulmonary diffusing capacity to nitric oxide and carbon monoxide during exercise and in the supine position: a test–retest reliability study
Source: Exp Physiol. 2023 Jan 9;108(2):307–17. doi: 10.1113/EP090883 (PMC10103891; doi:10.1113/EP090883)
Supplement: Supplementary file 3 — Supplemental File 2 [file EPH-108-307-s001.pdf]

Online Supplemental File 2

	**Upright rest** Group A + B (n=20)			**Exercise** Group A (n=11)			**Supine position** Group B (n=9)		
	**SRD (units)**	**CV (%)**	**ICC (fraction)**	**SRD (units)**	**CV (%)**	**ICC (fraction)**	**SRD (units)**	**CV (%)**	**ICC (fraction)**
**$D_{LCOcAdj}$** (mmol/ (min·kPa))	1.6 [1.2, 2.4]	5.7 [3.9, 6.3]	0.96 [0.92, 0.98]	2.4 [1.7, 4.1]	7.2 [6.0, 8.1]	0.95 [0.85, 0.98]	2.1 [1.5, 3.2]	6.1 [4.9, 7.0]	0.97 [0.88, 0.99]
**D_{MAdj}** (mmol/ (min·kPa))	9.9 [7.9, 13.0]	8.3 [7.3, 9.2]	0.95 [0.89, 0.98]	43.1 [29.4, 71.9]	18.4 [15.1, 20.8]	0.84 [0.51, 0.95]	5.4 [3.7, 8.8]	5.0 [4.0, 5.7]	0.98 [0.92, 0.99]
**V_{cAdj}** (mL)	16.4 [12.9, 21.9]	8.3 [7.3, 9.2]	0.89 [0.77, 0.95]	21.6 [14.9, 35.2]	10.4 [8.6, 11.7]	0.86 [0.58, 0.95]	30.7 [21.7, 48.3]	10.9 [8.8, 12.4]	0.88 [0.59, 0.97]
**V_{AAdj}** (L)	0.27 [0.18, 0.48]	1.6 [1.4, 1.7]	1.00 [0.99, 1.00]	0.26 [0.19, 0.38]	1.2 [1.0, 1.4]	1.00 [0.99, 1.00]	0.26 [0.17, 0.48]	1.6 [1.3, 1.8]	1.00 [0.99, 1]

Supplementary table: Reliability of $D_{LCO/NO}$ metrics corrected for O_2.

Data are presented with 95% CI [LL, UL]. **Abbreviations:** D_{LNO}, pulmonary diffusing capacity for nitric oxide; D_{LCOc}, pulmonary diffusing capacity for carbon monoxide corrected for haemoglobin; D_M, membrane diffusing capacity; V_c, pulmonary capillary blood volume; V_A, alveolar volume; SRD, smallest real difference; CV, coefficient of variation; ICC, intraclass correlation coefficient.
